# Visual Tuning May Boost African Cichlid Diversity

**DOI:** 10.1371/journal.pbio.1000267

**Published:** 2009-12-22

**Authors:** Robin Meadows

**Affiliations:** Freelance Science Writer, Fairfield, California, United States of America

**Figure:**
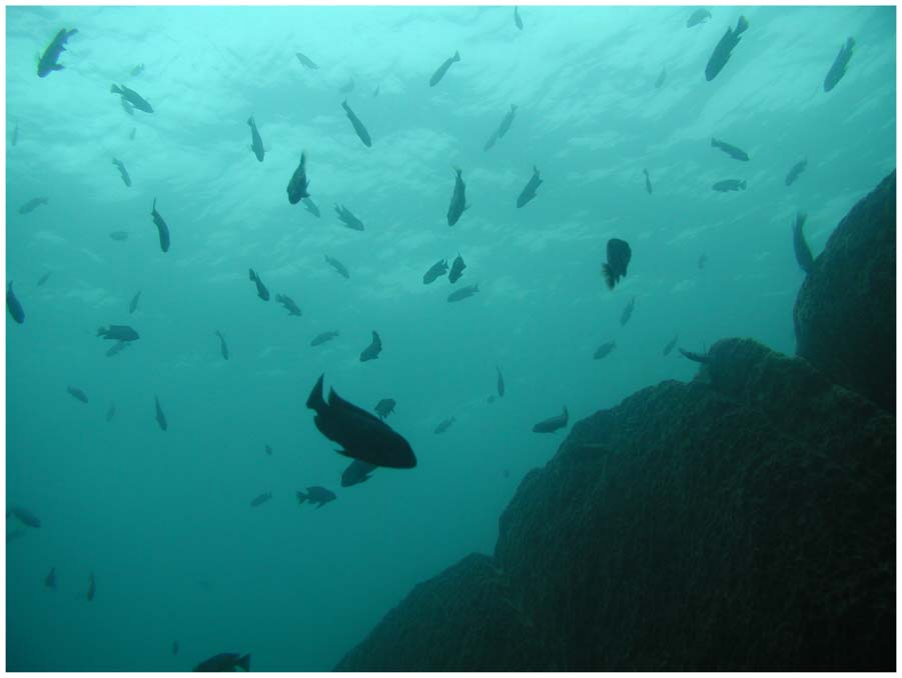
African cichlids are some of the most diverse fish on the planet, having radiated into several thousand species during the past few million years. They use visual cues to recognize conspecifics based on species-specific color patterns. Changes in gene regulation and coding sequence contribute to sensory diversification in two independent radiations of these cichlid fishes. Above: A cloud of cichlids illuminated by the bright downwelling light found in the clear waters of Lake Malawi (Image: Christopher Hofmann).

African cichlid fish form new species faster than any other vertebrates, with hundreds of species evolving within the last 2 million years in Lake Malawi and within the last 120,000 years in Lake Victoria. This rapid speciation makes cichlids good models for elucidating the genetic mechanisms behind biodiversity. Vision may play a key role in cichlid evolution, adapting them to forage for new foods or colonize new habitats. Vertebrate retinas have two groups of light-sensitive proteins called opsins: those in rod photoreceptors, which are sensitive to dim light, and those in cone photoreceptors, which are sensitive to color. Changes in the visual system could be due to differences either in the expression of opsin genes or in their DNA sequences. A Research Article in this issue of *PLoS Biology* by Christopher Hofmann and colleagues suggests that both mechanisms underlie changes in visual sensitivity in cichlids.

To investigate differences in cichlids’ visual sensitivity, the researchers compared 56 types of fish from Lake Malawi, where the water is spectacularly clear, and 11 types of fish from Lake Victoria, where the water is turbid. First, they used messenger RNA to compare the expression of opsin genes in the Malawi and Victoria fish. Cichlids have one rod opsin and six cone opsins, which range in sensitivity from ultraviolet to red wavelengths (roughly 300 to 700 nanometers). In addition, cichlids have two kinds of cones: single cones, which contain the three opsins that are sensitive to shorter wavelengths (ultraviolet, violet, and blue); and double cones, which contain the three opsins that are sensitive to longer wavelengths (blue-green, green, and red).

The results showed that Malawi cichlids collectively expressed all six cone opsins and so were sensitive to wavelengths across the spectrum. The researchers then used opsin expression patterns in Malawi cichlids to estimate the sensitivities of their single cones, which contain the shorter wavelength opsins, and double cones, which contain the longer wavelength opsins. They found that Malawi cichlids clustered into three distinct groups that are sensitive to short, middle, and long wavelengths. This opsin diversity is likely driven by foraging. For example, Malawi cichlids that eat other fish generally expressed more long-wavelength opsins, while those that eat plankton and algae generally expressed more short-wavelength opsins. Ultraviolet-sensitivity is known to increase fishes’ foraging efficiency on zooplankton and other microorganisms, suggesting that these differences in opsin expression may be adaptive.

In contrast, while all of the Victoria cichlids expressed the red-sensitive opsin, none of them expressed the ultraviolet-sensitive opsin. This makes sense because little ultraviolet light penetrates turbid water. However, expression of the violet-sensitive opsin varied amongst Victoria cichlids. The diversity of this opsin is likely driven by light levels, which vary by nearly four orders of magnitude in Lake Victoria’s murky waters. When the researchers compared opsin expression in cichlids from three parts of the lake ranging from relatively clear to relatively turbid, they found that fish from the clearer waters expressed more of the violet-sensitive opsin.

Next, the researchers compared the structures of opsins in cichlids from the two lakes. Based on DNA sequences, there were more functionally significant amino acid variations in the active regions of ultraviolet- and red-sensitive opsins, which are at the edges of cichlids’ visual range. Such amino acid substitutions have been shown to shift opsin sensitivities by 3–15 nm in fish.

Taking all of their findings together, the researchers propose that changes in gene expression generate large-scale shifts in opsin sensitivity of about 30–100 nm within cichlids’ visual range, while changes in DNA sequences fine-tune opsin sensitivity by about 5–10 nm at the extremes of their visual range. In turn, this resulting opsin diversity likely contributes to the tremendous diversity of African Rift Valley lake cichlids, which is estimated at 1,300 species. By changing the way cichlids see each other and their environment, large shifts in opsin wavelength sensitivity could change their mating and foraging behaviors and ultimately produce new species.

**Hofmann CM, O’Quin KE, N. Marshall NJ, Cronin TW, Seehausen O, et al. (2009) The Eyes Have It: Regulatory and Structural Changes Both Underlie Cichlid Visual Pigment Diversity. doi:10.1371/journal.pbio.1000266**

